# Development of an artificial intelligence model to estimate psychiatrist-assessed mental health–related presenteeism

**DOI:** 10.1093/joccuh/uiag055

**Published:** 2026-09-21

**Authors:** Shotaro Doki, Masakazu Hirokawa, Taiga Noguchi, Daisuke Hori, Soma Nishimura, Katsuya Hotta, Yuya Iwata, Naoko Kouda, Shota Matsumoto, Kenji Suzuki, Shin-ichiro Sasahara

**Affiliations:** Institute of Medicine, University of Tsukuba, 1-1-1 Tennodai, Tsukuba, Ibaraki 305-8575, Japan; Minds in Co., Ltd., 1-9-10 Nihonbashi Horidomecho, Chuo-ku, Tokyo 103-0012, Japan; Minds in Co., Ltd., 1-9-10 Nihonbashi Horidomecho, Chuo-ku, Tokyo 103-0012, Japan; Data Science Laboratories, NEC Corporation, 1753 Shimonumabe, Nakaharaku, Kawasaki, Kanagawa 211-8666, Japan; Center for Cybernics Research, University of Tsukuba, 1-1-1 Tennodai, Tsukuba, Ibaraki 305-8575, Japan; Graduate School of Science and Technology, University of Tsukuba, 1-1-1 Tennodai, Tsukuba, Ibaraki 305-8575, Japan; Institute of Medicine, University of Tsukuba, 1-1-1 Tennodai, Tsukuba, Ibaraki 305-8575, Japan; Graduate School of Comprehensive Human Sciences, University of Tsukuba, 1-1-1 Tennodai, Tsukuba, Ibaraki 305-8575, Japan; Retail & Consumer Service Division, Sojitz Corporation, 2-1-1 Uchisaiwaicho, Chiyoda-ku, Tokyo 100-8691, Japan; Human Capital Department 2, Sojitz Corporation, 2-1-1 Uchisaiwaicho, Chiyoda-ku, Tokyo 100-8691, Japan; Public Relations Department, Sojitz Corporation, 2-1-1 Uchisaiwaicho, Chiyoda-ku, Tokyo 100-8691, Japan; Digital Business Division, DG COMMUNICATIONS Co, Ltd, 3-7-30 Nishishinjuku, Shinjuku-ku, Tokyo 160-0023, Japan; Institute of Systems and Information Engineering, University of Tsukuba, 1-1-1 Tennodai, Tsukuba, Ibaraki 305-8575, Japan; Institute of Medicine, University of Tsukuba, 1-1-1 Tennodai, Tsukuba, Ibaraki 305-8575, Japan; Research and Development Center for Lifestyle Innovation, University of Tsukuba, 1-1-1 Tennodai, Tsukuba, Ibaraki 305-8575, Japan

**Keywords:** artificial intelligence, presenteeism, mental health, avatar, performance

## Abstract

**Objectives:**

This study aimed to contribute to the development of an AI-based system that supports worker health and productivity by enabling early detection of presenteeism. We tested whether an AI model could assess mental health–related presenteeism with accuracy comparable to that of psychiatrists and whether the frequency of application use was comparable between avatar-based and real-person interfaces.

**Methods:**

This study aimed to design a multivariable prediction model among white-collar employees in a Japanese company. The participants comprised 117 white-collar workers who provided a total of 1631 video responses to a standardized health-status question over a period of 10 working days. The primary outcome measure was the accuracy of the AI model in estimating workers’ mental health–related presenteeism. The secondary outcome was the frequency of application use when inquiring about workers’ health conditions, comparing the avatar-based interface with the real-person interface.

**Results:**

The AI model achieved an overall accuracy of 72.2%, a macro F1 score of 0.64, and a weighted F1 score of 0.73 compared with psychiatrists’ ratings. Agreement between the 2 psychiatrists was 84.5%. Participants were allocated to either an avatar-based interface or a real-person interface, with no significant differences observed between groups in baseline characteristics or frequency of application use, whereas a significant difference was observed in the frequency of missing values.

**Conclusions:**

The developed AI model demonstrated performance comparable to that of psychiatrists in estimating mental health–related presenteeism from video data. This approach offers a novel, objective alternative to traditional questionnaire-based methods.

## Teaser

**Key points**
 

- **What is already known on this topic:** Presenteeism is a major but largely invisible contributor to productivity loss, closely associated with workers’ mental and physical health, and its assessment has so far relied mainly on self-report questionnaires that have inherent limitations such as reporting bias and delayed detection.
- **What this study adds:** We developed an AI model for estimating mental health–related presenteeism from video data that achieved an accuracy of 72.2% and a macro F1 score of 0.64 in 3-class classification. The numbers of video responses and free-description entries did not differ between avatar and real-person interfaces.
- **How this study might affect research, practice, or policy:** The present model provides a scalable, objective tool for early detection of reduced work performance. It may complement existing occupational mental health practices by enabling frequent self-monitoring, and its interface flexibility may facilitate broader implementation while maintaining worker privacy.

## 1. Introduction

In recent years, the mental health of workers has attracted considerable social concern. Poor mental health not only leads to direct consequences such as absenteeism and resignation, but also manifests as “presenteeism,” a state in which employees attend work but are unable to perform adequately because of physical or psychological difficulties. Presenteeism is defined as the opposite of absenteeism.^1^ A cross-sectional study conducted in 2000 assessed presenteeism by asking: *Has it happened over the previous 12 months that you have gone to work despite feeling that you really should have taken sick leave due to your state of health?* The higher the frequency, the greater the level of presenteeism was considered to be.^2^

Presenteeism, being a largely invisible issue, is prone to delayed recognition and intervention, and is therefore regarded as a major factor contributing to reduced productivity. Recent studies have further demonstrated that presenteeism is associated with multiple factors, including mental health, physical health status, and environmental conditions in the workplace.^3^^,^^4^

From an economic perspective, the losses attributable to presenteeism are considerable and cannot be ignored. Previous studies have reported that productivity losses due to presenteeism exceed those caused by absenteeism.^5^^,^^6^ It has also been shown that employees with greater presenteeism experience greater losses than those with lesser presenteeism.^7^ Minimizing such losses is therefore an urgent priority for corporate management.

Early visualization of presenteeism would allow intervention prior to the progression of ill health. Within occupational health practice in particular, this may create a critical opportunity to provide appropriate support before the onset of mental health disorders. Establishing such a framework for early detection and intervention necessitates the development of methods capable of identifying workers at a premorbid stage, before their condition deteriorates.

To date, the screening of presenteeism has typically relied on questionnaire-based approaches.^2^^,^^8^^,^^9^ However, questionnaires present several limitations. Respondents’ self-assessments are prone to reference bias, whereby evaluations are influenced by surrounding norms or social group standards.^10^ In workplace stress surveys, defensive biasing also tends to increase when respondents perceive a lack of anonymity, leading them to underreport stress or related problems.^11^

To the best of our knowledge, no objective method for detecting presenteeism other than questionnaire-based approaches has been developed. However, attempts have been made to establish objective detection methods for stress—a concept closely related to presenteeism—using smart devices. Tani et al^12^ identified 11 technological categories under development, including those relevant to mental health such as sleep estimation, emotion analysis, self-care/cognitive behavioral therapy/mindfulness, psychological safety, and electroencephalographic devices. For example, one study reported that 2 distinct groups of patients with depression could be classified with 79% accuracy using voice features,^13^ while another showed that scores on the 9-item Patient Health Questionnaire could be predicted from video data, with a mean absolute error of 3.08.^14^ However, it has been noted that many of these findings may be attributable to confounding factors rather than the underlying grouping characteristics, and should therefore be interpreted with caution; hence, the accuracy cannot be considered sufficient.^15^

Although approaches not reliant on questionnaires are beginning to be developed, the available evidence remains insufficient. Therefore, this study aimed to develop an artificial intelligence (AI) model capable of automatically estimating workers’ presenteeism from objective video data, without depending on subjective self-reports.

In recent years, services that employ AI to deliver cognitive behavioral therapy or use avatars in virtual reality environments to conduct counseling have emerged.^16^ However, it remains unclear whether workers’ emotional expression differs depending on whether the interactive character within an application is presented as a real human or an avatar. Some studies have reported that the strength of the therapeutic alliance between a client and an avatar can be comparable to that between 2 humans^17^^,^^18^; however, to what extent workers would use the application when an avatar is employed in the interface compared with a real human remains unclear. Therefore, further investigation is required.

These technologies make it possible to detect the early signs of presenteeism during routine observation and avoid missing the appropriate timing for intervention. Ultimately, the present study aimed to contribute to the development of a system that supports both worker health and productivity. Given this background, the hypotheses tested in the present study were as follows:

1. An AI model would achieve a level of accuracy in assessing mental health–related presenteeism comparable to that of psychiatrists.
2. When inquiring about workers’ health conditions through the application, the frequency of use would be comparable between the avatar-based and real-person interfaces.

Presenteeism may result from a wide range of health conditions, including both physical disorders (such as allergic rhinitis and musculoskeletal disorders) and mental health problems. In this study, presenteeism was specifically defined as mental health–related presenteeism, based on psychiatrists’ evaluations of participants’ mental health status and its impact on work functioning.

## 2. Methods

### 2.1 Data collection

The study participants were recruited from a white-collar company. In a private booth within the company, the potential subjects used a PC terminal to complete questions on basic characteristics (age, sex, highest educational attainment, job position, working hours, commuting time, and income), as well as on sleep, emotions (Russell’s circumplex model), and performance (the Single-Item Presenteeism Question [SPQ]). Russell’s circumplex model evaluates emotional states by plotting affect along the following 2 axes on a 2-dimensional plane: pleasure–displeasure and arousal–sleepiness.^19^ The SPQ is a scale that measures presenteeism, defined as the degree to which work performance declines when continuing to work despite ill health.^20^ Higher scores indicate greater presenteeism, that is, poorer performance. On the PC terminal, the participants were asked “How are you feeling today?” via a video prompt, and their video-recorded responses were collected. The timing of the data collection was as follows: basic attributes were obtained only once at the initial session, whereas data on sleep, emotions, performance, and video recordings were collected on weekdays over 10 days between March and November 2022. Participants were asked to provide responses at least twice a day—typically upon arriving at and leaving work—but no strict enforcement was applied.

### 2.2 Randomization

Participants were randomly allocated into 2 groups using a random number table: one in which the interviewer asking about health status was presented as an avatar, and the other in which the interviewer was a real person. Both the avatar and real-person conditions used pre-recorded videos; therefore, real-time interaction was not possible. The randomization procedure using the random number table was conducted by individuals not involved in the data analysis. Because the participants were aware of whether an avatar was used, the study employed a single-blind randomization design. The avatar employed was the “Robot” provided by VRChat Inc (www.vrchat.com). This avatar is a gray robot with an oval-shaped head, simple eyes and mouth, a rounded torso with spherical joints, slender arms, and hands with 5 fingers. While it does not display facial expressions, its hands and head are capable of movement. In the real-person group, the interviewer was modeled on a Japanese male in his 30s. For both the avatar and real-person conditions, the upper body was shown so that hand movements could be observed.

### 2.3 Ground truth: psychiatrists’ assessments

The psychiatrists reviewed the collected data on basic attributes, mental health–related performance, and video recordings, and categorized each case as “Blue: Presenteeism Good,” “Yellow: Presenteeism Caution,” or “Red: Presenteeism Risk.” “Blue” indicated a state in which the worker was judged to have little to no decline in productivity; “Yellow” suggested an expected decline in productivity of mild to moderate degree; and “Red” represented a state in which a clear impact on work productivity was observed.

Psychiatrists were instructed to assess participants’ current condition at the time of recording by integrating information from both the video responses and self-reported measures, including SPQ scores, sleep status, and emotional state. The SPQ was not used as an independent measure of presenteeism; instead, it served as supplementary information to assist the psychiatrists’ evaluations.

For training data, the video data were used as the input, and the psychiatrists’ evaluations served as the ground truth labels. The ratings were performed by 2 psychiatrists independently: Psychiatrist A (S.D.), who holds qualifications as a government-authorized psychiatrist, occupational physician, and board-certified specialist/supervisor in social medicine; and Psychiatrist B (D.H.), who holds the same qualifications in addition to being a board-certified occupational health consultant. For discordant cases, S.D. and D.H. discussed them; if the disagreement could not be resolved, the cases were reviewed by S.S., an occupational physician, board-certified specialist in social medicine, and board-certified psychiatrist.

### 2.4 Statistical analysis

Machine learning was performed using gradient-boosted decision trees, implemented with LightGBM. Validation was conducted through leave-one-participant-out cross-validation. This method is a cross-validation approach in which 1 sample is held out for evaluation while all remaining samples are used for training, and the process is repeated iteratively until every sample has been used for evaluation.^21^ Features included facial expressions (facial action units derived from OpenFace), voice characteristics (intonation, pitch, response latency, and speech content), and body movements (head tilt). The performance of the AI model was compared with psychiatrists’ assessments, and accuracy, sensitivity, specificity, and weighted Cohen kappa were calculated. Differences in emotional expressions between the avatar and real-person conditions were examined using the Mann-Whitney *U* test for age, daily working hours, commuting time, sleep duration, number of responses to video prompts, and number of entries in the free-description field. Sex, highest education, job position, and income were compared using Pearson chi-square test. The frequency of missing values was compared between the avatar-based and real-person interfaces using a generalized linear mixed model (GLMM), with participant ID included as a random intercept to account for repeated measurements within participants. Part of this study design was prospectively registered in a public database (UMIN000046625). In this article, we report a secondary analysis using measures that were collected separately from the primary outcomes registered for the main study. Statistical analyses were conducted using Python and IBM SPSS Statistics for Windows (Version 30; IBM, Armonk, NY, USA).

### 2.5 Sample size

Because this was an exploratory study, no a priori sample size calculation was performed. Using G*Power version 3.1.9.7, we calculated the minimum detectable effect size for a 2-tailed *t-*test with a significance level of .05 and a statistical power (1 − β) of .80.

### 2.6 Ethical considerations

Informed consent was obtained from all participants. During the informed consent process, participants were informed that they would not suffer any disadvantage by choosing not to participate in the study, and that they could withdraw their consent at any time. Although the collected data were anonymized, complete anonymity could not be guaranteed for the video data, as the participants’ faces were visible. This study was designed in accordance with the ethical principles of the Declaration of Helsinki and approved by the Ethics Committee of the Faculty of Medicine, University of Tsukuba (approval No.: 1662).

### 2.7 Patient and public involvement

Patients and members of the public were not involved in the design, conduct, analysis, interpretation, reporting, or dissemination plans of this research. Members of the public participated solely as study participants, and no patient or public involvement (PPI) activities were undertaken.

## 3. Results

Responses were obtained from 117 participants, whose basic characteristics are shown in Table 1. The mean age (SD) of the participants was 36.8 (10.9) years. In terms of sex, 85 participants (72.6%) were male and 32 (27.4%) were female. The most common level of education was university graduation, reported by 86 participants (73.5%). Forty participants (34.2%) held managerial positions. The most frequent income range was 4.0-7.9 million yen, reported by 37 participants (31.6%). The mean (SD) number of daily working hours was 9.5 (3.6), the mean (SD) 1-way commuting time was 44.2 (16.9) minutes, and the mean (SD) daily sleep duration was 6.2 (0.9) hours.

**Table 1:** Participants’ characteristics (*N* = 117).

Characteristics	Mean	SD	*n*	%
**Age, y**	36.8	10.9	–	–
**Female sex**	–	–	32	27.4
**Education**				
**Technical college**	–	–	1	0.9
**University**	–	–	86	73.5
**Graduate school**	–	–	30	25.6
**Managerial position**	–	–	40	34.2
**Annual income (million yen)**				
**<4.0**	–	–	13	11.1
**4.0-7.9**	–	–	37	31.6
**8.0-11.9**	–	–	21	18.0
**12.0-15.9**	–	–	31	26.5
**≥16.0**	–	–	15	12.8
**Working hours/day**	9.5	3.6	–	–
**Commuting time, min**	44.2	16.9	–	–
**Sleep time, h**	6.2	0.9	–	–

A total of 1631 video responses were obtained (22 missing), with the number of responses per participant ranging from 1 to 29 (mean 12.5, SD 7.1). Psychiatrists’ assessments used as the ground truth were as follows: Psychiatrist A classified 1232 responses as blue (75.6%), 330 as yellow (20.2%), and 69 as red (4.2%); Psychiatrist B classified 1251 as blue (76.7%), 269 as yellow (16.5%), and 111 as red (6.8%). The agreement rate between psychiatrists A and B was 84.5%, and the weighted kappa coefficient was 0.661 (*P* < .001). The total number of datasets actually used for training and validation was 795. A total of 836 missing values were identified (349 due to audio-related issues, 97 due to image-related issues, and 390 due to other issues). Missing data attributable to audio processing occurred primarily because no participant speech was recorded or because the participant’s speech overlapped with the audio of the questions, making it difficult to isolate the participant’s voice. Most image-related issues attributable to facial recognition occurred because the participant’s face was not fully captured within the camera frame; for example, in some cases, only the forehead or upper portion of the face was visible. This was mainly due to the absence of a standardized procedure for adjusting the computer or seating position according to the participant’s body size. Other missing data were excluded during the feature calculation process based on criteria such as maximum and minimum values and threshold-based processing.

The accuracy of the AI model compared with psychiatrists’ ratings was 72.2%. The macro F1 score and weighted F1 score were 0.64 and 0.73, respectively (Figure 1). Sensitivity, specificity, and F1 score were as follows: blue versus yellow and red (sensitivity 77.2%, specificity 72.6%, and F1 score 0.82); yellow versus blue and red (sensitivity 58.4%, specificity 80.1%, and F1 score 0.51); and red versus blue and yellow (sensitivity 66.1%, specificity 95.5%, and F1 score 0.60).

**Figure 1:**
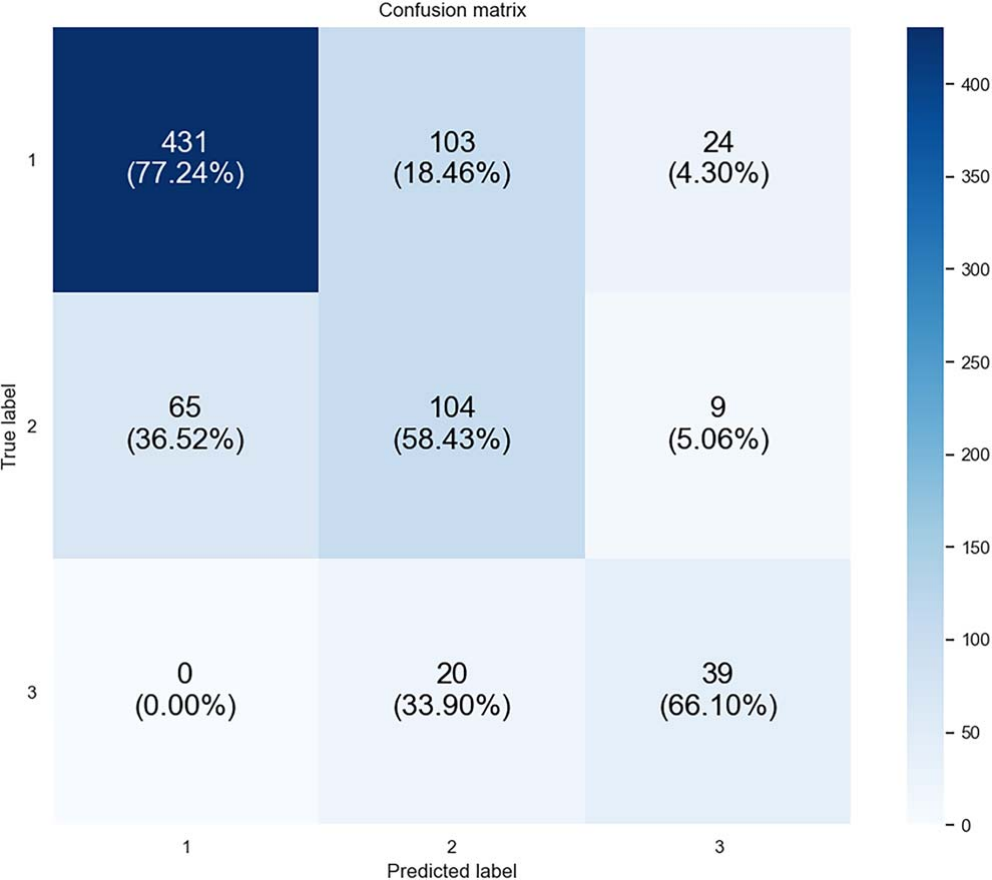
Confusion matrix. The true label represents the psychiatrists’ assessments, while the predicted label represents the outcomes determined by the AI model. On both the vertical and horizontal axes, 1 indicates “blue: good presenteeism,” 2 indicates “yellow: caution,” and 3 indicates “red: high risk.”

The SPQ scores were significantly correlated with both psychiatrists’ assessments (*r* = 0.135, *P* = .018) and the AI model’s assessments (*r* = 0.174, *P* = .002).

Participants were then allocated to an avatar group (*n* = 61; 52.1%) or a real-person group (*n* = 56; 47.9%) (Table 2). No significant differences with respect to baseline characteristics were observed between the 2 groups. Likewise, no significant differences in the numbers of video responses or entries in the free-description field were found (*P* = .266 and *P* = .981, respectively). A GLMM with participant ID included as a random intercept showed that the real-person interface was associated with significantly higher odds of successful video recognition than the avatar interface (odds ratio [OR] = 1.84, *P* < .001).

**Table 2:** Participants’ characteristics in the avatar and real-person groups.

	Avatar (*N* = 61)				Real person (*N* = 56)				
Characteristics	Mean	SD	*n*	%	Mean	SD	*n*	%	*P*
**Age, y**	37.8	11.0	–	–	35.8	10.9	–	–	.235
**Female sex**	–	–	16	35.6	–	–	16	28.6	.776
**Education**	–	–	–	–	–	–	–	–	.211
**Technical college**	–	–	1	0.9	–	–	0	0.0	–
**University**	–	–	41	73.5	–	–	45	80.4	–
**Graduate school**	–	–	19	25.6	–	–	11	19.6	–
**Managerial position**	–	–	21	34.4	–	–	19	33.9	.955
**Annual income (million yen)**	–	–	–	–	–	–	–	–	.212
**<4.0**	–	–	3	4.9	–	–	10	17.9	–
**4.0-7.9**	–	–	22	36.1	–	–	15	26.8	–
**8.0-11.9**	–	–	10	16.4	–	–	11	19.6	–
**12.0-15.9**	–	–	17	27.9	–	–	14	25.0	–
**≥16.0**	–	–	9	14.7	–	–	6	10.7	–
**Working hours/day**	9.9	4.8	–	–	9.1	1.2	–	–	.271
**Commuting time, min**	45.5	17.6	–	–	43.0	16.2	–	–	.459
**Sleep time, h**	6.2	0.9	–	–	6.2	0.8	–	–	.963
**No. of video responses**	13.2	7.4	–	–	11.7	6.7	–	–	.266
**No. of comment entries**	1.9	3.5	–	–	1.9	4.2	–	–	.981

Continuous variables were analyzed using the Mann-Whitney *U* test; categorical variables using Pearson chi-square test.

Using G*Power, the minimum detectable effect size (Cohen *d*) for the *t-*test was calculated assuming an α error of .05 and a statistical power (1 − β) of .80, resulting in an effect size of *d* = 0.52.

## 4. Discussion

In this study, we developed an AI model to estimate worker performance as assessed by psychiatrists. Unlike conventional stress checks, this approach enables the rapid detection of daily presenteeism, suggesting potential utility for self-care. In addition, no differences in the participants’ frequency of participation in the experiment or the extent of their free-description responses were observed, regardless of whether the interviewer’s video featured an avatar or a real person, whereas a significant difference was observed in the frequency of missing values.

In Japan, it is common for companies to assess the mental health of their employees. The Japanese government mandates that companies conduct mental health surveys at least once a year. Companies are not permitted to access individual results, but rather, analyze data at the group level to inform mental health initiatives in the workplace.^22^ According to a survey conducted in Japan, among those who had used digital mental health services, 69.2% reported using services provided by their employers.^23^ By utilizing the present model, employees can perform mental health self-checks not only once a year, but also on a weekly or monthly basis. This enables appropriate self-management that is less affected by fluctuations such as busy or off-peak work periods or temporary changes in stress levels.

The AI model developed in the present study demonstrated an accuracy of 72.2%, a sensitivity ranging from 58.4% to 77.2%, and a specificity from 72.6% to 95.5%. The interrater agreement between physicians was 84.5%, indicating that the model’s diagnostic precision was not yet equivalent to that of human experts. However, when compared with the accuracy of other medical devices using imaging or physiological data in the mental health field, 72.2% accuracy is not considered unsatisfactory. For example, near-infrared spectroscopy has been reported to achieve an accuracy of 74.6% in diagnosing depression.^24^ Furthermore, in reports on sleep-tracking devices such as Oura Ring, Fitbit, and Apple Watch, sensitivity has ranged from 61.7% to 79.5%, and precision from 50.5% to 87.8%.^25^ These values are comparable to those obtained with the AI model developed in the present study. The false-positive rate for the red category was relatively high at 45.8%, indicating that the model may identify a larger number of individuals as requiring attention. This could increase organizational costs due to additional follow-up assessments or interventions. However, the high negative predictive value of 97.2% is a major strength of this study.

Although numerous mental health assessment applications are commercially available, few studies have reported high-level evidence regarding their effectiveness. A systematic review published in 2025 reported on 4 self-monitoring–type mental health applications. That review found that while some studies demonstrated beneficial effects—such as reductions in hospitalization and relapse rates—and reported high adherence and retention, concerns about privacy, insufficient clinical support, and distrust in technology were identified as factors hindering long-term use.^26^ In the present study, the average number of responses was 12.5 out of the required 20 (62.5%), indicating that the adherence rate was not high. This may be due to the fact that the application only allowed users to record data without providing any feedback on their results. If feedback functionality were added, adherence rates could potentially improve.

During the study briefing, participants asked questions about how their data would be handled and how their privacy would be protected. In mental health management applications, it may be necessary to implement measures that alleviate such user concerns. A report comparing health management application policies across 9 countries identified common approval requirements, including transparency and safety. However, it also noted that issues such as oversupply, reimbursement, safety, and privacy remain significant, emphasizing the need for international regulatory harmonization.^27^ In Japan, employers are legally required to conduct an annual mental health assessment (“stress check”) for employees. Although employers cannot view individual results, they may utilize aggregated data, which are anonymized to protect privacy, for workplace improvement purposes. Similarly, the present system was designed to ensure employee privacy by allowing only aggregated data to be used by companies, in the same manner as the stress check. Video data were stored securely on encrypted servers managed by the research team, and employers cannot access individual-level data, identifiers, or original videos.

In this study, no differences in the participants’ frequency of participation in the experiment or their free-description responses were observed. One possible explanation for this is that the avatar was modeled after a robot with limited facial expressions, whereas the real-person version featured a male model in his 30s. Previous studies have shown that the stronger the human-likeness of an avatar, the more likely it is to elicit negative emotional reactions during emotion recognition tasks, whereas attributes such as “cuteness” or moderate nonhuman features tend to promote positive responses.^26^ However, it should be noted that several limitations, including the higher frequency of missing values in the avatar-based interface, may have introduced bias into the results, and that the present findings were obtained under limited conditions and therefore should not be generalized.

This study does have some limitations. First, although psychiatrists’ assessments of mental health–related presenteeism were used as the ground truth for developing the AI-based presenteeism estimation model, the validity and reliability of these psychiatrist evaluations were not verified. A major limitation of this study is that actual declines in work performance were not assessed, and therefore it remains unclear whether presenteeism in its conventional sense was actually measured. For future work, we aim to develop a program focused on coping with presenteeism. In addition, although this application is intended for use in occupational health settings, careful consideration must be given to its implementation and operation—particularly in relation to organizational policies on application use and the handling of personal information.

In this study, psychiatrists evaluated participants’ momentary mental health–related work performance states by integrating multiple sources of information, including video responses, working hours, sleep condition, emotional state, and SPQ scores. To make psychiatrists’ evaluations more robust and reliable, we intended to improve the quality of the ground truth labels by integrating multidimensional information. Therefore, the labels used in the present study should not be interpreted as evaluations derived solely from video data, but rather as reflecting comprehensive assessments integrating both behavioral and self-reported information.

Because the psychiatrists evaluated video recordings that could potentially allow identification of individual participants, the possibility of rater bias arising from repeated assessments of the same participant cannot be completely excluded. Although psychiatrists were instructed to evaluate each recording independently based on the participant’s observable condition at the time of recording, this potential bias may have influenced the assessments.

Presenteeism was evaluated by psychiatrists and therefore reflected a mental health perspective in this study. However, presenteeism can arise not only from mental health problems but also from physical conditions such as allergic rhinitis (hay fever) and musculoskeletal disorders. Nagata et al^6^ reported that the economic burden associated with musculoskeletal disorders among Japanese workers is comparable to that of mental health conditions. Therefore, it should be noted that the present study did not assess overall presenteeism but rather focused specifically on mental health–related presenteeism.

As for future perspectives, we plan to conduct validation studies with different populations (eg, other companies, industries, or age groups) to verify whether the model’s accuracy can be replicated and its reproducibility ensured. To improve participation rates, further refinements in the user interface/user experience design of the application will also be necessary. In addition, we aim to conduct intervention studies using this application to evaluate the effectiveness of stress monitoring. While paying careful attention to the handling of personal information, we also plan to perform organizational analyses to explore how the system can contribute to enhancing organizational productivity.

## Data Availability

Deidentified data and code may be available for educational or research purposes upon reasonable request to the principal investigator S.D. (doki.s@md.tuksuba.ac.jp), subject to interinstitutional data sharing agreements under the condition of approval of the Ethics Committee of the Faculty of Medicine, University of Tsukuba.
